# App-Based Tracking of Self-Reported COVID-19 Symptoms: Analysis of Questionnaire Data

**DOI:** 10.2196/21956

**Published:** 2020-09-09

**Authors:** Martin Zens, Arne Brammertz, Juliane Herpich, Norbert Südkamp, Martin Hinterseer

**Affiliations:** 1 Department of Medicine Kliniken Ostallgaeu-Kaufbeuren Fuessen Germany; 2 DESIGN-IT GmbH Frankfurt Germany; 3 University Medical Center Freiburg Freiburg Germany

**Keywords:** COVID-19, self-reporting, symptom, tracking, app, surveillance, distribution, digital tool, screening

## Abstract

**Background:**

COVID-19 is an infectious disease characterized by various clinical presentations. Knowledge of possible symptoms and their distribution allows for the early identification of infected patients.

**Objective:**

To determine the distribution pattern of COVID-19 symptoms as well as possible unreported symptoms, we created an app-based self-reporting tool.

**Methods:**

The COVID-19 Symptom Tracker is an app-based daily self-reporting tool. Between April 8 and May 15, 2020, a total of 22,327 individuals installed this app on their mobile device. An initial questionnaire asked for demographic information (age, gender, postal code) and past medical history comprising relevant chronic diseases. The participants were reminded daily to report whether they were experiencing any symptoms and if they had been tested for SARS-CoV-2 infection. Participants who sought health care services were asked additional questions regarding diagnostics and treatment. Participation was open to all adults (≥18 years). The study was completely anonymous.

**Results:**

In total, 11,829 (52.98%) participants completed the symptom questionnaire at least once. Of these, 291 (2.46%) participants stated that they had undergone an RT-PCR (reverse transcription-polymerase chain reaction) test for SARS-CoV-2; 65 (0.55%) reported a positive test result and 226 (1.91%) a negative one. The mean number of reported symptoms among untested participants was 0.81 (SD 1.85). Participants with a positive test result had, on average, 5.63 symptoms (SD 2.82). The most significant risk factors were diabetes (odds ratio [OR] 8.95, 95% CI 3.30-22.37) and chronic heart disease (OR 2.85, 95% CI 1.43-5.69). We identified chills, fever, loss of smell, nausea and vomiting, and shortness of breath as the top five strongest predictors for a COVID-19 infection. The odds ratio for loss of smell was 3.13 (95% CI 1.76-5.58). Nausea and vomiting (OR 2.84, 95% CI 1.61-5.00) had been reported as an uncommon symptom previously; however, our data suggest a significant predictive value.

**Conclusions:**

Self-reported symptom tracking helps to identify novel symptoms of COVID-19 and to estimate the predictive value of certain symptoms. This aids in the development of reliable screening tools. Clinical screening with a high pretest probability allows for the rapid identification of infections and the cost-effective use of testing resources. Based on our results, we suggest that loss of smell and taste be considered cardinal symptoms; we also stress that diabetes is a risk factor for a highly symptomatic course of COVID-19 infection.

## Introduction

COVID-19 was initially characterized as an acute respiratory infection with significant virulence and mortality. Following reports of the first cases in Wuhan, China, in December 2019, the virus spread globally with 9,738,374 confirmed cases as of June 26, 2020 [1]. Previous research has revealed that COVID-19, which is caused by the novel coronavirus SARS-CoV-2, is a systemic disease rather than an isolated acute respiratory illness [2,3]. The diversity in symptoms is the result of SARS-CoV-2 attacking various organs.

The aim of this study was to identify further symptoms and to investigate whether certain symptoms may be used as for screening to differentiate COVID-19 infection from other diseases. A powerful and reliable clinical screening tool may help to identify and isolate SARS-CoV-2–infected individuals and slow down disease prevalence.

## Methods

### Study Setting and Participants

**Figure 1:**
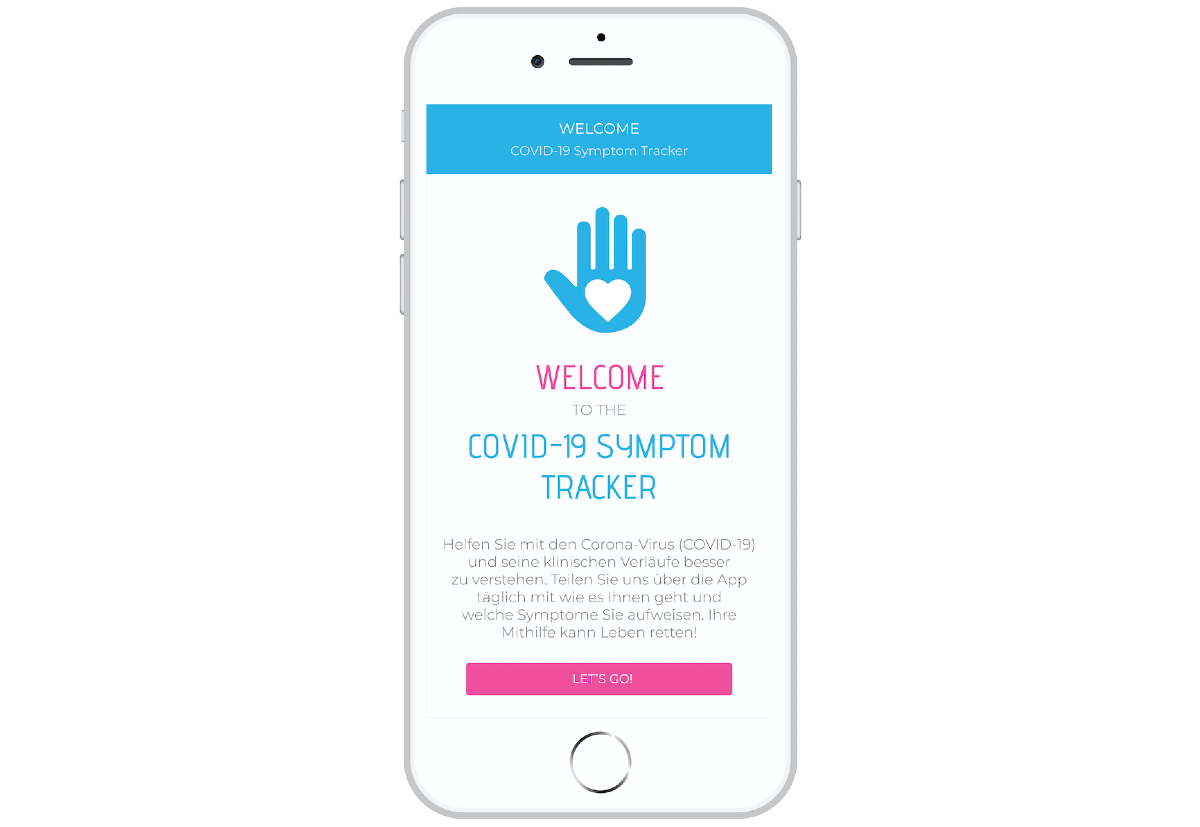
The COVID-19 Symptom Tracker.

The COVID-19 Symptom Tracker was developed by DESIGN-IT GmbH, in collaboration with the University Medical Center Freiburg and Kliniken Ostallgaeu-Kaufbeuren, Fuessen Hospital (Figure 1). The first version was released for Apple iOS on April 8, 2020, and for Google Android on April 20, 2020. Within 5 weeks, the app was downloaded 22,327 times. The app was released in German, English, French, and Spanish and advertised through public media (TV, radio, newspaper, online), following a nationwide press release. An initial questionnaire asked for demographic information (age, gender, postal code) and past medical history with relevant chronic diseases. The participants are notified daily by push notifications to report whether they were experiencing symptoms and if they had been tested for SARS-CoV-2. In addition to a binary response regarding the prevalence of symptoms, for some items (eg, fever, cough) we collected additional details like temperature range or expectoration. If users sought professional health care advice (eg, visited a hospital, a private practice, etc), additional questions regarding diagnostics and treatment were asked. Participation was open to all adults (≥18 years). The study was anonymous; a formal consent for participation was not required due to this fact but was asked for regardless.

### Statistical Analysis

Data were downloaded from the database server. Only data sets of individuals who live in Germany and had completed the entire symptom questionnaire at least once were included in further analysis. Participants were presented as counts, age as mean and standard deviation derived from an age span, and questionnaire items as counts and percentages. For each risk factor and symptom, odds ratios were calculated using binary logistic regression. Data from untested participants were not included in this analysis.

### Ethics

Approval of the study design was obtained by the Ethics Committee at the University Medical Center Freiburg (EK 337/20). The committee was very supportive of the project.

### Data and Code Availability

The anonymous data collected by the COVID-19 Symptom Tracker app can be shared with researchers upon request with a research protocol or due to a question of public interest, if permitted by the ethics committee. The app code is also available upon request. Requests can be sent to the corresponding author. The Laravel PHP script for SQL data extraction is publicly available [4].

## Results

Between April 8 and May 15, 2020, 22,327 individuals installed the COVID-19 Symptom Tracker on their mobile device. In total, 11,829 (52.98%) participants completed the symptom questionnaire at least once. Of these, 291 (2.46%) stated that they had undergone an RT-PCR (reverse transcription-polymerase chain reaction) test for SARS-CoV-2; 65 (0.55%) reported a positive test result and 226 (1.91%) a negative one. The mean number of reported symptoms in the group of untested participants was 0.81 (SD 1.85). On average, individuals with a negative result reported 4.26 symptoms (SD 2.52) and those with a positive result reported 5.63 symptoms (SD 2.82). All participants were asked to fill out an initial questionnaire on intrinsic risk and demographic data. The self-reported prevalence of risk factors (eg, diabetes: 917/11,538, 7.95%; hypertension: 3268/11,538, 25.19%; and smoking: 3679/11,538, 31.89%) showed a significant correlation with the data provided regularly by the German Federal Office of Statistics [5] and the Ministry of Health [6] (eg, diabetes: 7.2%; hypertension: 24.6%), which suggests that the study cohort is representative of the German population (age group: 18-65 years). Elderly individuals are underrepresented due to the methodology used in this study.

**Figure 2:**
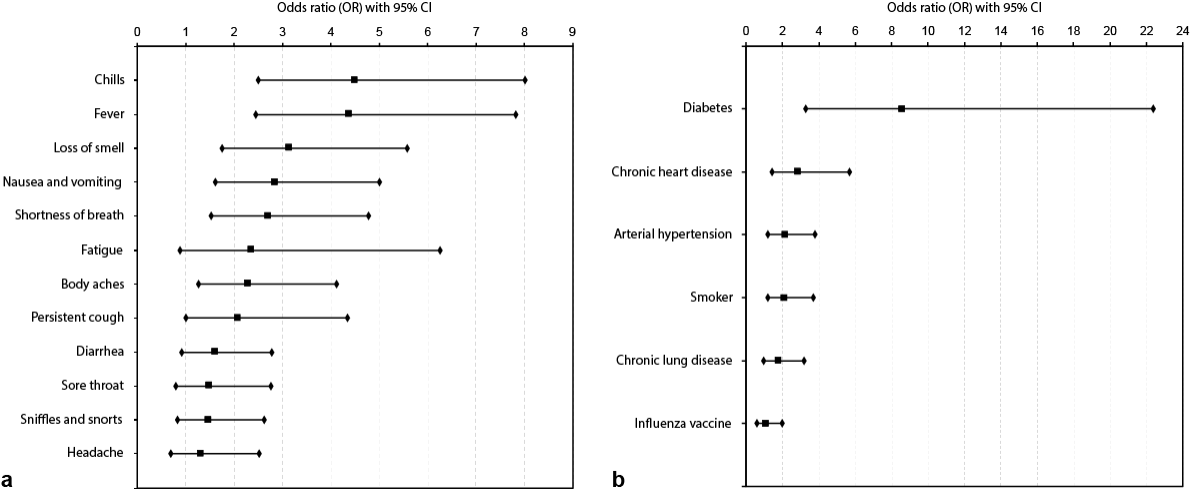
(a) Association between reported symptoms and the odds ratio for a positive SARS-CoV-2 test result; (b) association between reported risk factors and the odds ratio for a positive SARS-CoV-2 test result. Both analyses were carried out in a population of 291 participants who tested via reverse transcription-polymerase chain reaction. Error bars represent 95% CI.

The daily symptom questionnaire requested information on known or suggested symptoms as of April 1, 2020 [7-10] and permitted users to enter additional symptoms as free text. Odds ratios (OR) were calculated for all risk factors and reported symptoms. According to our data, the most significant risk factors are diabetes (OR 8.95, 95% CI 3.30-22.37) and chronic heart disease (OR 2.85, 95% CI 1.43-5.69) (Figure 2). We identified chills, fever, loss of smell, nausea and vomiting, and shortness of breath as the top five strongest predictors for a COVID-19 infection (Figure 2). The OR for loss of smell was 3.13 (95% CI 1.76-5.58); of comparable significance were chills (OR 4.48, 95% CI 2.51-8.01) and fever (OR 4.37, 95% CI 2.44-7.81). Chills, fever, and shortness of breath have been identified and communicated as common symptoms in the mainstream media. Nausea and vomiting (OR 2.84, 95% CI 1.61-5.00) has been reported as an uncommon symptom; however, our data suggest a significant predictive value. The characteristics of the entire cohort are displayed in Table 1.

**Table 1:** Characteristics of participants (N=11,829).

Characteristic		Tested for SARS-CoV-2		Not tested for SARS-CoV-2
		Positive test result	Negative test result	
Participants, n (%)		65 (0.55)	226 (1.91)	11,538 (97.54)
Female, n (%)		33 (50.77)	109 (48.23)	4360 (37.79)
Age (years), mean (SD)		42.65 (13.33)	41.04 (12.88)	44.47 (15.41)
Smoker, n (%)		33 (50.77)	74 (32.74)	3679 (31.89)
Influenza vaccine, n (%)		21 (32.31)	69 (30.53)	3268 (28.32)
**Comorbidities, n (%)**				
	Diabetes	14 (21.54)	7 (3.10)	917 (7.95)
	Arterial hypertension	26 (40.00)	54 (23.89)	2906 (25.19)
	Chronic lung disease	22 (33.85)	51 (22.57)	1352 (11.72)
	Chronic heart disease	17 (26.15)	25 (11.06)	1020 (8.84)
**Symptoms, n (%)**				
	Loss of smell	31 (47.69)	51 (22.57)	325 (2.82)
	Fatigue	60 (92.31)	189 (83.63)	1560 (13.52)
	Shortness of breath	40 (61.54)	84 (37.17)	554 (4.80)
	Fever	36 (55.38)	50 (22.12)	296 (2.57)
	Persistent cough	55 (84.62)	164 (72.57)	1290 (11.18)
	Diarrhea	34 (52.31)	92 (40.71)	670 (5.81)
	Chills	38 (58.46)	54 (23.89)	393 (3.41)
	Headache	50 (76.92)	162 (71.68)	1388 (12.03)
	Sniffling and snorting	43 (66.15)	129 (57.08)	1339 (11.61)
	Nausea and vomiting	34 (52.31)	63 (27.88)	428 (3.71)
	Body aches	45 (69.23)	112 (49.56)	1388 (12.03)
	Sore throat	48 (73.85)	148 (65.49)	1185 (10.27)

## Discussion

### Principal Findings

The COVID-19 pandemic has triggered numerous research endeavors that have focused on procuring a better understanding of the disease caused by the novel SARS-CoV-2. Agile and dynamic projects led to contemporaneous approaches with similar and comparable study designs. Menni et al [11] report of real-time symptom tracking tool to predict COVID-19 infections. The approach is based on a smartphone app available in the United Kingdom and United States; 2,618,862 participants were included and analyzed in order to design a prediction model. Simultaneously, a total of 22,327 participants reported potential COVID-19 symptoms on a smartphone-based app in Germany. However, unlike the cohort discussed in this work, the British cohort did not represent the general population (eg, overrepresentation of female participants).

Menni et al [11] suggested that loss of smell and taste should be included in routine screening for COVID-19. We strongly agree with this opinion as our data also suggest a strong predictive value. Although chills and fever are found in COVID-19 patients with a higher probability, in a clinical setting a loss of smell and taste is unique and allows a better differentiation from other infectious diseases since many are accompanied by chills and fever. In the present situation, we suggest that loss of smell and taste, especially in combination with other symptoms, ought to be considered a red flag and should result in immediate testing for SARS-CoV-2 as well as isolation of the patient until the test result is obtained.

Furthermore, our work suggests that an increased awareness of gastrointestinal symptoms, namely nausea and vomiting, is needed as these have a stronger predictive value for a COVID-19 infection than symptoms such as sore throat or persistent cough, which are commonly considered as typical.

Apart from that, a significant association was seen between every symptom mentioned in this paper and a positive test result for SARS-CoV-2. This finding is explained by the preselection of symptoms that were reported to be associated with COVID-19 in prior publications.

Manual screening of the answers to the open question which asked for additional symptoms revealed an accumulation of the following symptoms: vertigo, painful ears or eyes, burning sensation of the tongue, and thoracic pain. A static analysis of these reported symptoms is not yet possible due to insufficient data but corresponding questions have been added to the questionnaire in an updated version of the smartphone app.

Diabetes was identified as a major risk factor for a symptomatic course of COVID-19 infection. Other studies reported an increased risk, rapid progression, and a worse prognosis in patients with diabetes mellitus [12,13]. The mechanism remains unclear and requires further investigation.

### Limitations

The limitations of this study are mainly due to the self-reporting nature of our methodology for data retrieval. The design does not allow for the verification of the reported symptoms or test results. Apart from that, the participants are not invited or preselected and may not represent the general population. The use of a smartphone device may have resulted in an underrepresentation of older adults. Another possible limitation is the small number of participants that had been tested for SARS-CoV-2 infection. A possible correlation between age, gender, and postal code (demographic information) and individual symptoms was not considered in the univariate analysis of symptoms.

### Conclusions

Self-reported symptom tracking may help to identify novel symptoms of COVID-19 and estimate the predictive value of certain symptoms [14]. This may aid in the development of reliable screening tools. Clinical screening with a high pretest probability allows for the rapid identification of infections and serves as a cost-effective use of testing resources. Our data stress the necessity for an awareness of loss of smell and taste as cardinal symptoms.

## Abbreviations

OR: odds ratio
RT-PCR: reverse transcription-polymerase chain reaction
